# Characteristics of Methadone Intoxicated Children Presenting to Emergency Department; a Cross Sectional Study

**DOI:** 10.22037/emergency.v5i1.18780

**Published:** 2017-10-13

**Authors:** Parvin Kashani, Saeed Safari, Hamidreza Hatamabadi, Ali Arhami Dolatabadi, Mohammad Manouchehrifar, Maryam Dokht Tabrizi

**Affiliations:** 1 Emergency Department, Loghmane Hakim Hospital, Shahid Beheshti University of Medical Sciences, Tehran, Iran.; 2 Emergency Department, Shohadaye Tajrish Hospital, Shahid Beheshti University of Medical Sciences, Tehran, Iran.; 3 Emergency Department, Imam Hossein Hospital, Shahid Beheshti University of Medical Sciences, Tehran, Iran.

**Keywords:** Poisoning, pediatric emergency medicine, methadone, accident prevention, toxicity tests, acute

## Abstract

**Introduction:**

Each year a large number of patients present to emergency departments (EDs) following accidental or intentional poisoning with methadone. This study was designed with the aim of demographic evaluation of methadone poisoning in children presenting to ED and proposing preventive measures to parents and the healthcare system.

**Methods:**

This cross sectional study was carried out on children under the age of 12 years presenting to ED of a poisoning referral center. Demographic characteristics of the child and parents, cause of poisoning, form of drug consumed, dose consumed, the symptoms of the child on admission, clinical examination, laboratory findings, and final outcome were recorded and reported using descriptive statistics.

**Results:**

179 cases were studied (59.2% boys). Cause of consumption was accidental in 175 (97.8%) cases and consumed drug dose was unknown in 53 (53.6%) cases. On admission 6 cases were in deep coma, 133 (74.3%) had miotic pupils, and 52 (29.1%) were affected with respiratory apnea and cyanosis. In 132 (73.8%) cases drugs were obtained from unapproved stores and form of drug consumed was syrup in 146 (81.6%) cases. 177 (98.9%) cases were discharged after 2 or3 days and 2 (1.1%) cases died.

**Conclusion:**

Based on the results of the present study, most cases of methadone poisoning were accidental, in children residing in poor and middle-class areas, with parents who had a low level of education and had obtained the drug from unapproved stores and stored it in improper containers or at improper places. Only 64.8% of the parents were educated regarding drug storage.

## Introduction

Methadone is increasingly being used as a treatment for opium addiction and each year many individuals present to emergency departments (EDs) following intentional or accidental poisoning with it (1, 2). Clinical symptoms of methadone poisoning include loss of consciousness, respiratory depression, miotic pupils and other systemic (digestive, cardiac and neural) symptoms (3, 4).

Unfortunately, improper storage of the drug and children being able to access it has resulted in a considerable increase in presentation of children to ED following methadone poisoning (5-7). 

In a study in Maryland, prevention of accidental poisoning in children was successful using resistant containers, yet this problem still remains (8). Shadnia et al. showed that most accidental methadone poisonings had occurred due to consumption of the syrup form of drug and especially in children under 12 years of age (9). 

In Iran, methadone is available as 5, 20, and 40 mg tablets and 5 mg/cc syrup. Sadly, high prevalence of addiction to opium in Iran and having methadone at home for treatment purposes, which makes it also accessible to the families of those being treated, have led to an increase in the number of pediatric cases presenting with accidental poisoning. Therefore, the present study was designed with the aim of demographic evaluation of methadone poisoning in children presenting to ED and proposing preventive measures to parents and the healthcare system.

## Methods

Study design and setting

This cross sectional study was carried out on children under the age of 12 presenting to poisoning emergency unit of Loghmane Hakim Hospital, Tehran, Iran, from April 2015 for 18 months. This hospital is the referral center for poisoning cases in Iran’s capital. The study was approved by the Ethics Committee of Shahid Beheshti University of Medical Sciences and the researchers adhered to ethical research practices based on Declaration of Helsinki and maintained confidentiality of patient data throughout the study period. While orally explaining the study protocol, informed consent was obtained from the patients’ parents for being included in the study.

***Participants***

All the children aged less than 12 years old with methadone poisoning who presented to the mentioned ED were evaluated. Cases with suspicion to other poisonings, using multiple medications simultaneously, without a confirmed diagnosis, and having missing data were excluded from the study.

***Data gathering***

Demographic characteristics of the child and parents (age and sex, educational level and occupation), cause of poisoning, form of drug consumed (tablet or syrup), dose consumed, history of addiction in parents, educations received in methadone maintenance therapy (MMT) clinics and their implementation by the parents were evaluated and gathered for all the patients via a pre-designed checklist. In addition, the symptoms of the child on admission, clinical examination findings, medical history, laboratory findings and final outcome (death, discharge) were also recorded for the patients. A senior emergency medicine resident was responsible for data gathering under the supervision of an emergency medicine specialist.

The impression of methadone intoxication was made based on history taking and clinical examination and the diagnosis was confirmed by laboratory testing.

*Statistical analysis*

Data were statistically analyzed using SPSS 21 software. To describe data, frequency and percentage or mean ± standard deviation were used.

## Results

***Baseline*** ***characteristics***

179 children with methadone poisoning diagnosis visited the mentioned ED during the study period (59.2% boys). Table 1 shows the baseline characteristics of the patients. 71 (39.7%) cases were the only child in the family and 173 (96.6%) of the patients were residents of poor or middle-class areas of the city. Cause of consumption was reported to be accidental in 175 (97.8%) cases and intentional in 4 (2.2%). Consumed drug dose was stated as unknown in 53 (53.6%) cases.

Clinical symptoms

On admission only 71 (39.7%) patients were fully conscious and 6 (3.3%) cases were in deep coma. 133 (74.3%) cases had miotic pupils and 52 (29.1%) were affected with respiratory apnea and cyanosis. Vomiting, itching, restlessness, ataxia, and drooping eyelid were detected in 29.5%, 13.9%, 1.67%, 1.11%, and 0.5% of the studied children, respectively.

Obtaining and storing the drug

In 132 (73.8%) cases drugs were obtained from unapproved stores and only in 47 (26.2%) cases from approved pharmacies. Form of drug consumed was syrup in 146 (81.6%) cases and tablet in 33 (18.4%) cases. 116 (64.8%) of the parents had been educated on storing methadone by MMT clinics and 101 (87.1%) claimed that they had taken the necessary precautions regarding drug storage. Place of storage was refrigerator in 67 (37.4%) cases and in other cases storage was done in other parts of the house accessible to children. Drug was kept in a proper container in only 32 (17.9%) cases and in other cases it was stored in unlabeled containers along with other medications.

Outcome

3 (1.67%) cases were discharged from the hospital after 2 days and 174 (97.2%) after 3 days of hospitalization in pediatrics department and naloxone prescription. 2 (1.1%) cases that were discharged against medical advice died. 6 (3.3%) of the cases encountered aspiration and 5 (2.8%) had seizure while, 4 (2.2%) needed cardiopulmonary resuscitation (CPR). Metabolic acidosis, respiratory acidosis and respiratory alkalosis were seen in 12.8%, 25.1% and 2.2%, respectively.

**Table 1:** Baseline characteristics of the studied children

Variable	Number (%)
**Child’s sex**	
Boy	109 (60.9)
Girl	70 (39.1)
**The child’s guardian**	
Parents	161 (89.9)
Others	18 (10.1)
**Area of residence**	
Poor	130 (72.6)
Middle-class	43 (24.0)
Wealthy	6 (3.4)
**Parents’ university degree**	
Mother	40 (22.3)
Father	39 (21.8)
**Parents’ occupation**	
Stay at home mother	127 (70.9)
Laborer father	133 (74.3)
**Time of reaching the hospital**	
Less than 1 hour	16 (8.9)
More than 1 hour	163 (91.1)

## Discussion

Based on the results of the present study, most cases of methadone poisoning were accidental, in children residing in poor and middle-class areas, with parents who had a low level of education and had obtained the drug from unapproved stores and stored it in improper containers or at improper places. Only 64.8% of the parents were educated regarding drug storage.

Most of the evaluated children (59.2%) in the present study were boys. In surveys by Ghorbani et al. (10), Sharif et al. (11) and Esmaeili et al. (7), also boys were predominant with 59.3%, 56.9% and 58.3% frequencies, respectively. In a study by Jabbehdari et al. (12), frequency of both sexes in methadone poisoning was the same. Meanwhile, in a study by Aghabiklooei et al. (13), girls were predominant (55.6%). 

In the present study, consumed drug dose was known in 65.4% of the cases. Mean methadone dose consumed in Aghabiklooei et al. study (13) was 130 mg, mean consumption was 33 mg for methadone syrup and 21mg for methadone tablets in Jabbehdari et al. study (12), and 30 mg methadone syrup in Sharif et al. study (11). 

Based on AVPU scale, more than half of the studied children (56.98%) were in a state of consciousness where they would respond when their parents talked to them in a loud voice and 39.66% were conscious. 3.35% of the evaluated children did not respond to any stimulation. The clinical symptoms observed in the studied patients were similar to other studies. In Ghorbani et al. study (10) the most common symptoms of methadone poisoning in children included: sleepiness (61.9%), loss of consciousness (36.4%), vomiting (30.9%), miotic pupils (29.9%), cyanosis (22.7%), ataxia (14.4%), vertigo (8.2%), drowsiness (7.4%), apnea (6.2%), hypoxia (5.2%) and respiratory distress (4.1%). In the children studied by Sharif et al. (11) the observed symptoms were: sleepiness (91.4%), miotic pupils (75.9%), vomiting (69%), apnea (53.4%), cyanosis (43.1%), seizure (8.6%) ataxia (6.9%) and hallucination (3.4%). Additionally, in the study by Jabbehdari et al. (12) the patients’ symptoms included: sleepiness (75%), miotic pupils (68%), vomiting (61%), and apnea (40%).

The results obtained from the current study showed that 35.2% of the studied families had not received the necessary trainings from MMT clinics. On the other hand, only about half of all those who had been trained (56.4%) had implemented the teachings.

Considering the increasing prevalence of methadone poisoning in children, this statistic is very worrying and shows the poor performance of MMT clinics and related organizations. MMT clinics are responsible for educating all the individuals who get methadone from them and instruct them to implement the teachings and inform them on the irreversible consequences of disregarding the teachings and not implementing the necessary points. In addition, considering the role of media such as radio and television in increasing the awareness of the general population, short teasers should be made and broadcasted from radio and television. 

In our study, prevalence of methadone poisoning was higher in children who had parents with lower levels of education and resided in poor areas of the city. Based on the findings of the present study, a large number of the evaluated families (73.2%) had obtained methadone from unapproved stores. Therefore, regulatory authorities should continuously and accurately monitor these unapproved stores as well as MMT clinics and ask these centers to print safety instructions on drug brochures and avoid selling the drug without giving the necessary information to individuals.

Improper storage of this substance, especially in the form of syrup in soft drink bottles, which can be mistaken for water due to its color and appearance, increases the risk of poisoning in children. In this study, according to the parents’ statements, 97.8% of the children had consumed methadone accidentally and in most cases (69.3%), the container has not been specified for the drug. Additionally, in 78.2% of the cases, the form of drug consumed was syrup. On the other hand, placing the tablet form of this drug in places within the reach of children (in out reports placing it on the kitchen counter, on the floor, on the table, etc.) increases the risk of the child eating it and being poisoned.

Among the other worrying statistics found in this study, it can be noted that in most of the methadone intoxicated children evaluated in this study (88.8%), more than 1 hour had passed from the presentation of symptoms when they reached the hospital. This can in turn lead to a raise in apnea and cyanosis cases and therefore, increase the risk of mortality.

In this evaluation of 179 children, 2 were discharged against medical advice and died. This statistic shows that the parents and relatives are not aware of the irreversible consequences of this type of poisoning in children or they are not responsible enough. There is a need for giving enough information to the parents and the children, based on their age, and laws should be passed in this regard and a committee should monitor the execution of these laws.

For obtaining more accurate and reliable results, it is suggested to reproduce the present study with a larger sample size in various cities all over Iran and at different times to reach a more accurate pattern regarding these poisonings in children. Then the corresponding authorities can design and implement an accurate and practical program in this regard by considering the needs of each area based on its findings.

## Conclusion

Based on the results of the present study, most cases of methadone poisoning were accidental, in children residing in poor and middle-class areas, with parents who had a low level of education and had obtained the drug from unapproved stores and stored it in improper containers or at improper places. Only 64.8% of the parents were educated regarding drug storage.
